# Familiarity with the experimenter influences the performance of Common ravens (*Corvus corax*) and Carrion crows (*Corvus corone corone*) in cognitive tasks^☆^

**DOI:** 10.1016/j.beproc.2013.11.013

**Published:** 2014-03

**Authors:** Lara Cibulski, Claudia A.F. Wascher, Brigitte M. Weiß, Kurt Kotrschal

**Affiliations:** a Core Facility Konrad Lorenz Forschungsstelle for Behaviour and Cognition, University of Vienna, 4645 Grünau, Austria; b Department of Behavioural Biology, University of Vienna, 1090 Vienna, Austria; c Department of Behavioural Biology, University of Münster, 48149 Münster, Germany; d Comparative Zoology Group, Institute of Evolution and Ecology, University of Tübingen, Germany

**Keywords:** Carrion crow, Cognition, Common raven, Familiarity, Human–animal relationships

## Abstract

When humans and animals interact with one another over an extended time span they familiarise and may develop a relationship, which can exert an influence on both partners. For example, the behaviour of an animal in experiments may be affected by its relationship to the human experimenter. However, few studies have systematically examined the impact of human–animal relationships on experimental results. In the present study we investigated if familiarity with a human experimenter influences the performance of Common ravens (*Corvus corax*) and Carrion crows (*Corvus corone corone*) in interactive tasks. Birds were tested in two interactive cognitive tasks (exchange, object choice) by several experimenters representing different levels of familiarity (long and short-term). Our findings show that the birds participated more often in both tasks and were more successful in the exchange task when working with long-term experimenters than when working with short-term experimenters. Behavioural observations indicate that anxiety did not inhibit experimental performance but that the birds’ motivation to work differed between the two kinds of experimenters, familiar and less familiar. We conclude that human–animal relationships (i.e. familiarity) may affect the experimental performance of corvids in interactive cognitive tasks.

## Highlights

• We compared the results of corvids in experiments with familiar/unfamiliar humans.
• We investigated behavioural reactions towards familiar and unfamiliar humans.
• Corvids performed significantly better in experiments with familiar humans.
• Corvids did not show more neophobia towards unfamiliar humans.
• Hence, familiarity positively affected the experimental performance of corvids.

## 1 Introduction

Human–animal relationships can have a strong emotional component similar to relationships between humans (Kotrschal et al., 2009; McNicholas et al., 2005). Companion animals may actually contribute to the well-being and even health of humans, for example by providing social support and by facilitating social contacts to other humans (Beetz et al., 2012; McNicholas et al., 2005; Prothmann, 2008). Likewise, humans considerably influence companion animals: stroking, for instance, can reduce acute stress responses in dogs (Hennessy et al., 1998). Kotrschal et al. (2009) found effects of the owner–dog relationship on stress hormone levels and working performance of human–dog dyads: owners, which were closely attached to their dogs, effectively provided social support to their dogs in stressful situations. Such effects do not seem to be limited to domesticated species. In Greylag geese (*Anser anser*), for instance, it has been shown that human foster parents provide social support as effectively as goose parents (Frigerio et al., 2003; Weiß and Kotrschal, 2004).

Human–animal relationships may develop whenever humans and animals are able to distinguish each other individually and interact with each other regularly, for example during care-taking activities (Bayne, 2002; Chang and Hart, 2002; Russow, 2002). The ability to recognise individual humans has already been shown in a variety of species including several species of corvids (pigeons, *Columba livia*: Belguermi et al., 2011; Common ravens, *Corvus corax*: Bugnyar, 2007; review on mammal and bird species: Davis, 2002; magpies, *Pica pica*: Lee et al., 2011; mockingbirds, *Mimus polyglottos*: Levey et al., 2009; American crows, *Corvus brachyrhynchos*: Marzluff et al., 2010; horses, *Equus caballus*: Sankey et al., 2010; Carrion crows, *Corvus corone corone*: Wascher et al., 2012). Furthermore, it is known that the neurology (Goodson, 2005) and physiology (DeVries et al., 2003) of social behaviour are highly conserved among vertebrates and that the same mechanisms for bonding are involved in humans, and some animals (Odendaal, 2000). During the familiarisation with individual humans stress and neophobia of animals are gradually reduced (Bayne, 2002; Chang and Hart, 2002; Russow, 2002). In agreement with this, von Bayern and Emery (2009) showed that jackdaws (*Corvus monedula*) took longer to retrieve hidden food in the presence of an unfamiliar human than in the presence of a familiar person. The reduction of neophobia may also increase the animals’ motivation to work and thereby facilitate experimental procedures (Davis, 2002). Therefore, the presence of individual humans (Bayne, 2002; Davis, 2002) or interactions between humans and animals (Odendaal, 2000) may ultimately influence scientific results, such as measurements of hormone concentrations or anxiety-like behaviours. It has even been assumed that human–animal relationships possibly affect the performance of non-human animals in cognitive tasks and that a positive relationship between the experimenter and the focal individual may have contributed to the discovery of impressive cognitive abilities - for example by Irene Pepperberg in Grey parrots (Davis, 2002). Recent evidence suggests that human–animal relationships may indeed play a role during certain experiments (Péron et al., 2012). While there may be nothing wrong with optimising test performance by maintaining optimal relationships with an experimental animal (Péron et al., 2012), the problem may rather be that animals may not cooperate with unfamiliar experimenters, thus showing suboptimal performance that would wrongly be interpreted as evidence for cognitive constraints in a certain individual/species. On the contrary, concerns have been expressed that human–animal relationships may interfere with experimenter objectivity (Schilhab, 2002) and could lead to “Clever Hans” phenomena (Miklósi and Soproni, 2005; Rosenthal, 1967).

Recent research has revealed impressive cognitive abilities in various corvid species (rooks, *Corvus frugilegus*: Bird and Emery, 2009; Common ravens, *C. corax*: Bugnyar, 2007; Western scrub-jays, *Aphelocoma californica*: Dally et al., 2004; New caledonian crows, *Corvus moneduloides*: Hunt, 1996; review on several corvid species: Seed et al., 2009). A number of these studies made use of interactive experiments, that is experiments which necessitate contact between the bird and the experimenter or which involve manipulations by the experimenter (Common ravens, *C. corax* and Carrion crows, *C. corone corone*: Dufour et al., 2012; Carrion crows, *C. corone corone*: Mikolasch et al., 2011; jackdaws, *C. monedula*: Schloegl, 2011; Clark's nutcrackers, *Nucifraga columbiana*: Tornick et al., 2010; Carrion crows, *C. corone corone*: Hoffmann et al., 2011; jackdaws, *C. monedula*: von Bayern and Emery, 2009). One of the reasons why working with corvids is challenging is their pronounced neophobia (Heinrich, 1988, 1999; Heinrich et al., 1995). Corvids are proven to be able to distinguish between individual humans (Common ravens, *C. corax*: Bugnyar et al., 2007; American crows, *C. brachyrhynchos*: Marzluff et al., 2010; Magpies, *Pica pica*: Lee et al., 2011; Carrion crows, *C. corone corone*: Wascher et al., 2012) and also show neophobic behaviour towards unfamiliar humans (Heinrich, 1999; von Bayern and Emery, 2009). Thus, interactive experiments with corvids require a certain familiarity with the experimenter. However, the impact of familiarity or human–animal relationships on the results of corvid cognition studies has never been investigated systematically. Therefore, the aim of our study was to provide insight into the effects of familiarity with the experimenter on corvid cognition research. Specifically, we tested the hypothesis that familiarity influences the behavioural response of corvids to experimenters and the experimental performance in interactive cognitive tasks. Several different experimenters representing different levels of familiarity conducted two interactive experiments, an exchange and an object choice task, with Common ravens (*C. corax*) and Carrion crows (*C. corone corone*). We predicted that the birds’ participation rates and performance would be positively affected by familiarity with the experimenter. In addition to the experiments, the behavioural reactions of the birds to the different experimenters were monitored. We expected that the animals would show more affiliative and less stress-related behaviours towards more familiar experimenters.

## 2 Methods

### 2.1 Study subjects

The study was conducted with five captive Common ravens (three males, two females; age 2–15) and seven captive Carrion crows (three males, four females; age 2–4) at the Konrad Lorenz Research Station (KLF) in Grünau im Almtal (Austria) between January and June 2011.

Birds were kept in outdoor aviaries and could be individually distinguished by coloured leg bands. Crows were kept as two pairs and one trio; ravens were kept as two pairs and two singles which were given the opportunity to pair during the course of the study. All birds were fed a mixed diet (meat, bread, fruit, vegetables, milk products) twice daily and water was available ad libitum for drinking and bathing. Birds were not food-deprived prior to the experiments. Except for one raven (a zoo-bred individual) and one crow (a wild bird that was injured and delivered to a shelter shortly after fledging) all birds were hand-raised. All birds regularly participated in different studies investigating their cognitive abilities. The birds were separated for the tests so that they could be tested individually. However, they always had visual and acoustic contact to the other bird(s).

### 2.2 Experimenters

In total, 12 experimenters participated in the present study (termed experimenters A–L in the following). All were female to avoid possible effects of sex differences in interaction style (Hennessy et al., 1998; Kotrschal et al., 2009; Wedl et al., 2010). Experimenters who had performed experiments with the birds and carried out feeding duties for at least two months were considered “long-term experimenters” (experimenters A–E). Note that experimenter D was a long-term experimenter for the ravens and a short-term experimenter for the crows as she had only worked with the ravens prior to this study. Long-term experimenters did not go through a special preparation phase prior to the experiments. In contrast, “short-term” experimenters were newly introduced to the birds for the present study (experimenters D, F, G, H, I, J, K, L). They had a seven-day habituation period with the birds and then performed experiments for four days. For the habituation the short-term experimenters approached the aviaries twice daily for approximately 5–10 min and moved around in front of the wire mesh.

Various studies indicate that clothes are not crucial for human individual recognition in birds (Belguermi et al., 2011; Lee et al., 2011; Levey et al., 2009; Marzluff et al., 2010). Nonetheless, to exclude any potential effects of different clothing (Heinrich, 1999; Rybarczyk et al., 2003) all experimenters wore an identical shirt and jeans during habituation and experiments. For the experiments all experimenters announced themselves saying “hallo” to the birds but subsequently conducted the experiments without talking to the birds any more. Due to logistical constraints some experimenters worked only with the crows, and testing with long and short-term experimenters could not be balanced across seasons (Table 1).

### 2.3 Experimental procedure

In both tasks, the birds performed two sessions of 10 trials each with each experimenter in pseudo-randomised order (note that experimenters worked during different seasons and experimenter order was thus not fully randomised; see Table 1). Half of the birds started with the exchange task and the other half started with the object choice task. Experiments were conducted in the morning (between 0800 h and 1200 h) and in the afternoon (between 1400 h and 1800 h). The birds were separated from their conspecifics prior to the experiment by LC whereas only the respective experimenter was present during the experiments. The experiments were conducted through the wire mesh (i.e. the experimenter was outside the aviary). The experiments were video-recorded and analysed by LC (participation: yes/no, performance: correct/incorrect). In addition, long-term experimenters noted the results on data sheets during the experiment to check for interobserver-reliability (accordance of the results from data sheets and video coding: 100%).

#### 2.3.1 Exchange task

Ravens and crows were previously trained to exchange non-preferred food for preferred food items (Dufour et al., 2012). This task involves a cost-benefit consideration by the bird which is presumably affected by the subject's motivation and the effort necessary to obtain the most valuable food item (Dufour et al., 2012). In a similar delayed gratification task children considered experimenter reliability when deciding whether to consume a small reward instantly or wait for a larger reward (Kidd et al., 2013). Thus, the birds’ behaviour in the exchange task could also be affected by whether they consider the experimenter to be a reliable exchange partner.

One piece of standard food (bread, approximately 1.0 cm × 1.0 cm × 0.7 cm for ravens, 0.7 cm × 0.7 cm × 0.5 cm for crows) and one piece of same size preferred food (cheese) were presented in the palms of the experimenter out of reach of the bird. The experimenter then passed the standard food to the bird. After the bird had taken the standard food, the experimenter waited for two seconds with the presenting hand closed to a fist to allow the bird to make a decision. The preferred food item remained visible in the other palm. Then the experimenter opened the fist so that the bird could return the standard food into the opened palm. If the bird returned the standard food it received the preferred food item. A trial was considered successful only if the bird took the standard food and returned it to the experimenter as described. An interruption of this process by eating or caching the standard food was considered a failure. If a bird did not take the standard food or if it did not approach the experimenter so that an interaction was not possible, this was considered non-participation and the experimenter continued with the next trial.

#### 2.3.2 Object choice task

In the object choice task the birds were given a cue by the experimenter to indicate food hidden in one of two cups. The study subjects had previously participated successfully in such tasks (Mikolasch et al., 2011). The experimenter knelt in front of the wire mesh outside the aviary. A piece of food (1/16 piece of commercial dog food for crows and 1/8 for ravens) was hidden below one of two red cups (1 cm high, diameter 4 cm) on a blue board (50 cm × 20 cm). Cups were placed approximately 25 cm apart. The food was hidden under the cups behind a blind (35 cm × 16 cm × 12 cm), out of view of the birds. The position of the reward (left/right) was randomised and both cups were rewarded equally often. After removing the blind the experimenter provided the bird with a cue about the location of the food by turning her head to look at the rewarded cup and touching it three times with the ipsilateral hand. Afterwards, the experimenter took her hand back, held it in a relaxed position close to her body and looked at the bird. After three seconds the board was moved towards the bird to allow it to choose a cup by touching it with the beak. Then the board was moved away from the bird again and the cups were turned over so that the bird could see under which cup the reward was hidden. If the choice was correct the bird received the food item. In case of a wrong choice the reward was placed in the middle of the blue board and the next trial was started. To attract a focal individual's attention in case it did not approach the setup or did not choose, the experimenter put her hands to the edges of the board, called the bird's name and placed her hands back towards her body. If the bird still did not approach or make a choice, this sequence was repeated. If the bird did not react to this twice, this was considered non-participation.

### 2.4 Behavioural observations

The behaviour of the birds towards the different experimenters was monitored outside of the experimental context. The experimenters approached the aviaries (in case of short-term experimenters after seven days of habituation) twice and moved along the aviary slowly at a distance of approximately 0.5 m to the wire mesh. The behaviour of the birds was video-recorded by LC for 5 min per approach and videos were coded with Solomon Coder beta 11.07.04 (Copyright by András Péter; http://solomoncoder.com) by LC. Special attention was paid to affiliative (“approach”), stress-related (“wing-quivering”, “fluttering”) and comfort behaviours (“preen”, “pluster”, “stretch”, “scratch”; Table S1, see supplementary data).

### 2.5 Statistical analysis

We performed generalised linear mixed models (GLMMs) with SPSS 19 for the dependent variables “participation” (yes/no) and “performance” (correct/incorrect) using a binomial logistic regression. In addition to “familiarity” we considered all factors which we think could have contributed to differences in participation and performance rates: the identity of each “person” (nested within familiarity) was included to investigate if potential differences were caused by individual-specific characteristics of the experimenter (by individual-specific characteristics we mean, e.g. body language, voice, hair colour, etc.). “Season” (divided into pre-breeding, breeding and post-breeding season, see Table 1) was included to check if seasonal effects, which are known to strongly affect bird behaviour (Helm et al., 2006; Nelson, 2005), contributed to potential differences. In addition the interactions species × familiarity and season × familiarity were included due to the unbalanced study design. “Animal identity” was included as a random term in all models to account for repeated measurements.

Due to the unbalanced participation of the different experimenters over the course of the study, the effects of “person” and “season” were confounded. Therefore, two separate analyses were performed containing either “season” (S models) or “person” (P models) as a fixed factor for each dependent variable. These models were compared by the Akaike Information Criterion corrected for small sample sizes (AICc), a measure of model accuracy (Garamszegi et al., 2009), whereby an AICc that is smaller by two or more units indicates a better model fit. The results of the parallel S and P models were similar: in most cases terms were significant in both models and the magnitude and direction of differences were similar. Since the S models had lower AICc values, tables and graphs in the following section show the results of S models complemented by additional results from P models.

We reduced the full models by stepwise exclusion of non-significant fixed terms, taking into consideration the AICc: if the exclusion of a non-significant term increased the AICc this term was re-entered into the model. Excluded terms were re-entered into the final model singly to confirm non-significance (Garamszegi et al., 2009). Pairwise comparisons were calculated for fixed factors with more than two factor levels and the method of least significant differences was used for post hoc corrections. Estimated mean values (EM) and standard errors (SE) are given for the factor levels. Estimated means differ from actual means in that they are calculated taking into consideration all factors included in the model. The advantage of this method is that the effects of multiple factors on the variable of interest are not treated singly but are considered simultaneously. We present estimated means rather than actual means to integrate all investigated factors in this manner. To assess the relative importance of significant fixed effects, effect sizes were taken into account: effects with effect sizes higher than one were considered strong effects, effect sizes of about 0.5 moderate and effect sizes below 0.5 were considered weak effects (Garamszegi et al., 2009).

In the object choice task both cups were rewarded equally often, so that the birds could choose correctly in 50% of the trials even if they chose cups randomly. Therefore we performed binomial tests on the performances of each bird with the different experimenters to test if the birds’ performance deviated from a chance level of 50% or 0.5, respectively. Additionally we calculated the percentage of correct trials for each bird and evaluated differences in performance between long and short-term experimenters using Wilcoxon signed-rank tests.

For the statistical analysis of behavioural observations only behaviours occurring in more than 10% of the approaches were used. The results were then transformed into binomial data (occurrence of the behaviour yes/no), summarised according to functional contexts (Table S1, see supplementary data) and analysed with GLMMs with the same parameters as the experimental results. For brevity's sake we only report significant terms that remained in the respective final model in the results. Full results are given in the tables (Tables 2–4).

## 3 Results

### 3.1 Exchange task

**Fig. 1:**
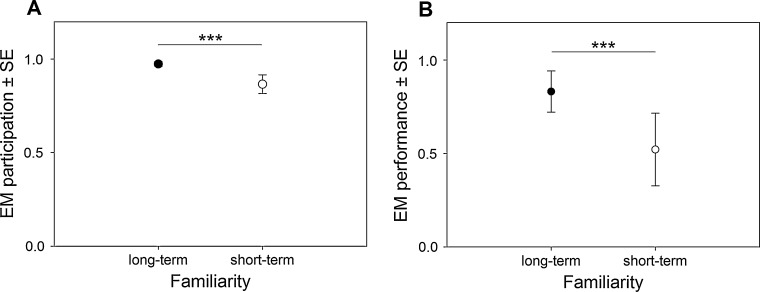
Estimated mean participation rates (A) and performance (B) ±SE in the exchange task with long-term -and short-term experimenters (“season” model data). ***p < 0.001.

Participation rates in the exchange task were significantly affected by familiarity: the birds participated more often in the experiments with long-term experimenters (EM_trials participated_ = 0.974 ± 0.013) than with short-term experimenters (EM_trials participated_ = 0.886 ± 0.050; Fig. 1A). Seasonal effects on participation rates were weak; the birds participated more often during the pre-breeding (EM_trials participated_ = 0.954 ± 0.022) and breeding (EM_trials participated_ = 0.969 ± 0.016) season than during the post-breeding season (EM_trials participated_ = 0.885 ± 0.051). The interaction species × familiarity remained in the final model, but pairwise comparisons did not show any significant results. Participation rates were higher with long-term experimenters during the pre-breeding (EM_trials participated_ = 0.985 ± 0.008) and breeding season (EM_trials participated_ = 0.990 ± 0.006) than during the post-breeding season (EM_trials participated_ = 0.890 ± 0.053). The birds participated more often in the task with long-term than with short-term experimenters during the pre-breeding (long-term experimenters: EM_trials participated_ = 0.985 ± 0.008; short-term experimenters: EM_trials participated_ = 0.866 ± 0.061) and breeding season (long-term experimenters: EM_trials participated_ = 0.990 ± 0.006; short-term experimenters: EM_trials participated_ = 0.907 ± 0.043). Person-specific effects were strong but not significant in pairwise comparisons (GLMM, Table 2). The lower AICc of the S model (12,165.907 as compared to an AICc of 16,187.614 in the P model) indicates that seasonal effects explain the observed differences better than person-specific effects.

There was also a strong effect of familiarity with the experimenter on performance: the birds exchanged food successfully more often in experiments with long-term (EM_correct exchanges_ = 0.831 ± 0.110) than with short-term experimenters (EM_correct exchanges_ = 0.521 ± 0.194; Fig. 1B). Seasonal effects on performance were strong and significant in pairwise comparisons: the birds performed significantly better during the pre-breeding (EM_correct exchanges_ = 0.838 ± 0.107) and the post-breeding (EM_correct exchanges_ = 0.789 ± 0.132) than during the breeding season (EM_correct exchanges_ = 0.391 ± 0.185). Ravens (EM_correct exchanges_ = 0.935 ± 0.072) performed significantly better than crows (EM_correct exchanges_ = 0.270 ± 0.197). Performance with long-term experimenters was higher during the pre-breeding (EM_correct exchanges_ = 0.875 ± 0.087) and post-breeding (EM_correct exchanges_ = 0.929 ± 0.055) than during the breeding season (EM_correct exchanges_ = 0.562 ± 0.195). Performance with short-term experimenters was significantly higher during the pre-breeding (EM_correct exchanges_ = 0.792 ± 0.132) than during the breeding (EM_correct exchanges_ = 0.243 ± 0.144) and post-breeding season (EM_correct exchanges_ = 0.514 ± 0.195). The birds performed worse with short-term experimenters during the breeding than during the post-breeding season. Significant differences between long-term and short term experimenters concerning performance occurred during the breeding (long-term experimenters: EM_correct exchanges_ = 0.562 ± 0.195; short-term experimenters: EM_correct exchanges_ = 0.243 ± 0.144) and post-breeding season (long-term experimenters: EM_correct exchanges_ = 0.929 ± 0.055; short-term experimenters: EM_correct exchanges_ = 0.514 ± 0.195) but not during the pre-breeding season (long-term experimenters: EM_correct exchanges_ = 0.875 ± 0.087; short-term experimenters: EM_correct exchanges_ = 0.792 ± 0.132), although the effect of familiarity was strongest during the pre-breeding season (GLMM, Table 3). Person-specific effects were strong and pairwise comparisons showed that the performance of the birds differed significantly between different experimenters (GLMM, Table 3). Again the AICc of the S model (AICc = 9221.836) was lower than the AICc of the P model (AICc = 9256.926), indicating a better fit of the S model.

### 3.2 Object choice task

**Fig. 2:**
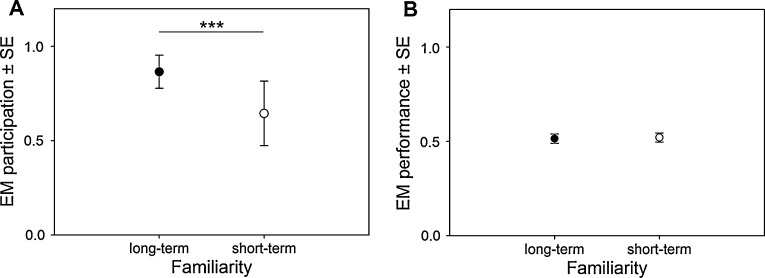
Estimated mean participation rates (A) and performance (B) ±SE in the object choice task with long-term and short-term experimenters (“season” model data). ***p < 0.001.

Familiarity had a strong effect on the birds’ participation rates: the birds participated more often in the object choice task when they were working with long-term experimenters (EM_trials participated_ = 0.865 ± 0.088) than when they were working with short-term experimenters (EM_trials participated_ = 0.644 ± 0.171; Fig. 2A). Season also had a strong effect, which remained significant in pairwise comparison: participation in the object choice task was significantly higher during the breeding (EM_trials participated_ = 0.944 ± 0.040) than during the pre-breeding season (EM_trials participated_ = 0.569 ± 0.185) and the post-breeding season (EM_trials participated_ = 0.638 ± 0.174). Ravens participated with long-term experimenters more often (EM_trials participated_ = 0.645 ± 0.275) than with short-term experimenters (EM_trials participated_ = 0.428 ± 0.291). Participation rates with long term experimenters were significantly higher during the pre-breeding (EM_trials participated_ = 0.813 ± 0.118) and breeding season (EM_trials participated_ = 0.978 ± 0.016) than during the post-breeding season (EM_trials participated_ = 0.568 ± 0.189). The birds participated significantly more often with short-term experimenters during the breeding (EM_trials participated_ = 0.862 ± 0.090) and post-breeding season (EM_trials participated_ = 0.702 ± 0.157) than during the pre-breeding season (EM_trials participated_ = 0.287 ± 0.157); participation rates with short-term experimenters were significantly higher during the breeding than during the post-breeding season. Participation rates with long-term experimenters and short-term experimenters differed significantly during the pre-breeding (long-term experimenters: EM_correct exchanges_ = 0.813 ± 0.118; short-term experimenters: EM_correct exchanges_ = 0.287 ± 0.157) and post-breeding season (long-term experimenters: EM_correct exchanges_ = 0.568 ± 0.189; short-term experimenters: EM_correct exchanges_ = 0.702 ± 0.157), although the effect of familiarity was strong during the breeding season as well (GLMM, Table 4). Person-specific effects were strong and pairwise comparisons showed that the participation rates of the birds differed significantly between different experimenters (GLMM, Table 4). Model fit of the S model (AICc = 14,194.300) was better than model fit of the P model (AICc = 14,240.664).

None of the fixed terms had a significant effect on performance in the object choice task (GLMM, Table S2, see supplementary data, Fig. 2B). A further analysis of performance revealed that only in a few cases the birds’ performance differed from chance level at all. Two crows performed above chance level with short-term experimenter G (binomial test, *p* = 0.002 and *p* = 0.0041, respectively). One crow performed significantly below chance level with short-term experimenter J (binomial test, *p* = 0.041). On a group level the birds’ performance did not differ from chance level with either long-term (Wilcoxon signed-rank test, *n* = 5, *Z* = −1.557, *p* = 0.120, Median 52.94% of trials correct) or short-term experimenters (Wilcoxon signed-rank test, *n* = 8, *T* = −0.178, *p* = 0.859, Median 49.38% of trials correct).

### 3.3 Behavioural observations

Concerning the birds’ behaviour towards the experimenters we found that only the frequency of approaches towards the experimenter differed significantly between long-term and short-term experimenters (GLMM, Table S3, see supplementary data): the birds approached long-term experimenters (EM_occurence_ = 0.304 ± 0.135) significantly more often than short-term experimenters (EM_occurence_ = 0.062 ± 0.034). There was a significant interaction of season and familiarity with long-term experimenters being approached less often during the pre-breeding (EM_occurence_ = 0.071 ± 0.060) than during the breeding season (EM_occurence_ = 0.713 ± 0.142). Long-term experimenters (EM_occurence_ = 0.713 ± 0.142) were approached significantly more often than short-term experimenters (EM_occurence_ = 0.034 ± 0.026) during the breeding season. The P model revealed that ravens (EM_occurence_ = 0.469 ± 0.165) approached experimenters significantly more often than crows (EM_occurence_ = 0.081 ± 0.044). Ravens (EM_occurence_ = 0.809 ± 0.132) tended to approach long-term experimenters more often than crows (EM_occurence_ = 0.139 ± 0.082). Ravens also tended to approach long-term experimenters more often than short-term experimenters (EM_occurence_ = 0.156 ± 0.089). The AICc of the P model was lower (AICc = 1168.727) than the AICc of the S model (AICc = 1208.150).

Ravens (EM_occurence_ = 0.692 ± 0.068) showed more comfort behaviour than crows (EM_occurence_ = 0.251 ± 0.046) and the occurrence of comfort behaviours differed between seasons: frequencies of comfort behaviour were significantly higher during the pre-breeding (EM_occurence_ = 0.625 ± 0.081) than during the breeding (EM_occurence_ = 0.382 ± 0.069) and post-breeding season (EM_occurence_ = 0.388 ± 0.062). Model fit of the P model (AICc = 984.934) was better than of the S model (AICc = 992.420). Familiarity, however, had no effect on the occurrence of comfort behaviours (GLMM, Tab. S4, see supplementary data). None of the fixed terms had a significant effect on the occurrence of stress-related behaviours (GLMM, Tab. S5, see supplementary data).

## 4 Discussion

For the first time we demonstrate effects of familiarity on the performance and behaviour of corvids in interactive cognitive tasks: birds participated more often in an exchange and in an object choice task when working with a long-term experimenter than when working with a short-term experimenter. In addition, the birds’ success rates in the exchange task were higher when working with long-term experimenters. We thereby provide evidence that familiarity may not only affect anxiety-like or stress-related behaviours as previously reported (von Bayern and Emery, 2009; Bayne, 2002; Chang and Hart, 2002) but also the outcome of interactive cognitive experiments. Success rates in the object choice task were not affected by familiarity with the experimenter.

During behavioural observations the birds did not show more stress-related behaviours towards short-term than towards long-term experimenters. This indicates that, unlike in other studies (von Bayern and Emery, 2009), the birds’ behaviour during the experiments is unlikely to be affected by the corvid-typical neophobia towards unfamiliar humans. These findings agree with other reports of neophobia reduction in the course of repeated interactions between humans and animals (Bayne, 2002; Chang and Hart, 2002; Russow, 2002). Therefore we assume, that the birds had habituated to short-term experimenters even within the comparably short time-span of one week, although we cannot completely exclude the possibility that the birds still were more nervous in the presence of short-term experimenters. Thus, we suggest that differences in the birds’ motivation to work, but not neophobia as described by von Bayern and Emery (2009), may account for the birds’ differential participation rates and performances when working with long or short-term experimenters.

Considering recent evidence for children's sensitivity towards experimenter reliability in a delayed gratification task (Kidd et al., 2013) it can be assumed that our subjects perceived long-term experimenters as more reliable exchange partners, either due to their shared experimental history or an existing human–animal-relationship: the birds did not only participate more often in both tasks and performed better in the exchange task but also approached long-term experimenters more often than short-term experimenters during behavioural observations. Comparable observations were made in studies on horses and were interpreted as an indication for the existence of a relationship between the animals and familiar experimenters (Sankey et al., 2010). In a review of his work Davis (2002) reports that even brief sociopositive interactions with a handler lead to a preference for contact with this handler in rats. These studies indicate that animals may develop preferences for familiar humans (Davis, 2002). We suggest that long-term experimenters and birds shared a relationship that affected the birds’ behaviour during the experiments, which is supported by the levels of affiliative behaviour the birds displayed towards long-term experimenters (Sankey et al., 2010). However, our results may also have been caused by an intermixture of the effects of familiarity and reinforcement history: long-term experimenters had regularly shared positive interactions with the birds, fed them and provided rewards during experiments in the past. This might have caused the birds to develop a preference for interactions with long-term experimenters (Davis, 2002). On the other hand, repeated positive interactions between humans and animals are known to promote the formation of human–animal relationships (Bayne, 2002; Chang and Hart, 2002; Russow, 2002). Currently, it is impossible to determine if our findings were caused by reinforcement history or by a relationship between the subjects and long-term experimenters. Therefore, in the future, the effect of reinforcement on the development of preferences and human–animal relationships needs to be investigated. Also, means to measure human–animal relationships, for example by quantifying affiliative behaviours and human–animal interactions (Sankey et al., 2010), might be useful to evaluate the relationship between individual experimenters and their subjects more precisely.

Notwithstanding, our findings demonstrate that familiarity may have considerable effects on the results of interactive cognitive experiments with corvids and may thus be of interest for corvid cognition research in general. Moreover, we assume that our results may have consequences for interactive work with other animals: in many institutions daily care-giving is conducted by different persons than the experimental procedures and thus familiarity between experimenters and animals may be reduced or lacking entirely although its beneficial effects (e.g. reduction of animals’ stress levels) are known (Bayne, 2002; Chang and Hart, 2002; Davis, 2002). Since several species have been shown to develop preferences for familiar humans (Davis, 2002), in the future the effects of familiarity should be considered more carefully not only in corvid research but in the behavioural sciences in general.

Surprisingly, the birds’ performance in the object choice task hardly deviated from chance level at all, although previous studies have clearly demonstrated the effects of touching an object on the choice decisions of juvenile ravens and Carrion crows via local enhancement (Mikolasch et al., 2011; Schloegl et al., 2008). Also, wild ravens use gestures to communicate (Pika and Bugnyar, 2011). Possibly the birds were not able to interpret the cue and did not perceive touching the cup as object manipulation as used in the studies of Mikolasch et al. (2011) and Schloegl et al. (2008). Alternatively, the birds may not have used the cue although they had understood the intention. Currently, we cannot specify the exact reason for the birds’ failure.

The birds’ participation and performance rates differed between different seasons.

The effect of season on experimental performance was not a main focus of the present study. However, due to the experimental design we were not able to collect all data in one seasonal phase and had to perform experiments during the breeding season, during which birds generally are not very motivated to participate in cognition experiments. Surprisingly, in our experiment participation rates were lower in the pre-breeding phase than during breeding and post-breeding. However, according to our expectations, performance was lower during the breeding season than during the pre- and post-breeding season. Seasonal effects could have been caused by hormonal (e.g. elevated levels of sex steroids) and behavioural changes (e.g. territory defence, courtship) accompanying breeding (Helm et al., 2006; Nelson, 2005). Ravens participated more often in the exchange task and were more successful in this task than crows; in contrast, ravens participated less often in the object choice task. The effect of familiarity with the experimenter, however, was not influenced by the species. Hence, neither species nor the unbalanced distribution of species across seasons can account for the present results. We also detected person-specific effects on participation rates and performance. Person-specific effects on human–animal-interactions were shown in a study on dogs (Kotrschal et al., 2009). These effects are assumed to be mediated by the owner's interaction style, which is affected by owner personality (Kotrschal et al., 2009). However, due to logistical reasons we could not take into account the experimenters’ personality or interaction style in the present study. Therefore, in the future it might be interesting to examine potential effects of these factors and to systematically vary the experimenters’ interaction style. Although effects of season and person were confounded to some extent and further studies will be needed to determine the importance of seasonal and experimenter-specific factors on corvids’ behaviour in interactive cognitive tasks, the effects of familiarity with the experimenter on participation rates and performance were robust.

In summary we show that the effects of familiarity between an experimenter and her subjects extend beyond a reduction of stress- and anxiety-like behaviours (Bayne, 2002; Davis, 2002) and that familiarity even affects the outcome of interactive cognitive experiments. In the future, these effects should be examined and considered more carefully not only in corvid research but in the behavioural sciences in general, as many species are theoretically capable of forming relationships with humans, which, in turn, can influence experimental results.

## Figures and Tables

**Table 1:** Testing schedules for Common ravens and Carrion crows. The level of familiarity (short-/long-term) is given for each experimenter.

	Pre-breeding		Breeding			Post-breeding	
	January	February	March		April	May	June
*Crows*							
Familiarity	Long	Short	Short		Long	Short	Long
Experimenter	A, B, C	D, F	G, H		E	I, J, K, L	C

*Ravens*							
Familiarity			Long	Short	Long	Short	Long
Experimenter			C, D	G, H	B, E	I, J, K, L	C

**Table 2:** Results of GLMMs with participation in the exchange task as the dependent variable. Statistical parameters for the final model are given as well as factors entered into the model, degrees of freedom (df), *F*- and *p*-values. *F*- and *p*-values of excluded factors were taken from full models. Effects sizes are given for terms that remained in the final model. The results shown originate from S models; results written in italics originate from models containing the fixed factor “person”.

Factor	df	*F*	*p*	Factor level	Effect size
Final model	7	25.168	<0.001		

Species	1	0.833	0.362	Crow	0.135
				Raven	1.590

Familiarity	1	43.350	<0.001	Long-term	−0.458
				Short-term	0

Season	2	24.933	<0.001	Pre-breeding	−0.132
				Breeding	0.284
				Post-breeding	0

Species × familiarity	1	5.236	0.022	Crow × long-term	1.101
				Raven × long-term	0
				Raven × short-term	0

Season × familiarity	2	22.604	<0.001	Pre-breeding × long-term	2.259
				Breeding × long-term	2.214
				Post-breeding × long-term	0
				Pre-breeding × short-term	0
				Breeding × short-term	0
				Post-breeding × short-term	0

*Person (familiarity)*	*11*	*9.971*	*<0.001*	*A*	*6.780*
				*B*	*6.628*
				*C*	*−2.171*
				*D*	*4.680*
				*E*	*0*
				*D*	*−1.785*
				*F*	*−2.215*
				*G*	*−2.339*
				*H*	*−0.484*
				*I*	*−2.294*
				*J*	*−1.980*
				*K*	*−2.679*
				*L*	*0*

**Table 3:** Results of GLMMs with performance in the exchange task as the dependent variable. Statistical parameters for the final model are given as well as factors entered into the model, degrees of freedom (df), *F*- and *p*-values. *F*- and *p*-values of excluded factors were taken from full models. Effects sizes are given for terms that remained in the final model. The results shown originate from S models; results written in italics originate from models containing the fixed factor “person”.

Factor	df	*F*	*p*	Factor level	Effect size
Final model	6	29.201	<0.001		

Species	1	5.607	0.018	Crow	−0.904
				Raven	2.765

Familiarity	1	73.751	<0.001	Long-term	2.524
				Short-term	0

Season	2	52.590	<0.001	Pre-breeding	1.285
				Breeding	−1.192
				Post-breeding	0

*Species* *×* *familiarity*	*2*	*2.857*	*0.058*	*Crow* *×* *long-tern*	*−3.108*
				*Raven* *×* *long-term*	*0*
				*Crow* *×* *short-term*	*−3.877*
				*Raven* *×* *short-term*	*0*

Season × familiarity	2	9.218	<0.001	Pre-breeding × long-term	−1.918
				Breeding × long-term	−1.139
				Post-breeding × long-term	0
				Pre-breeding × short-term	0
				Breeding × short-term	0
				Post-breeding × short-term	0

*Person (familiarity)*	*11*	*11.100*	*<0.001*	*A*	*2.282*
				*B*	*1.171*
				*C*	*1.744*
				*D*	*−0.025*
				*E*	*0*
				*D*	*1.743*
				*F*	*0.861*
				*G*	*−1.646*
				*H*	*−0.948*
				*I*	*0.175*
				*J*	*0.030*
				*K*	*−0.337*
				*L*	*0*

**Table 4:** Results of GLMMs with participation in the object choice task as the dependent variable. Statistical parameters for the final model are given as well as factors entered into the model, degrees of freedom (df), *F*- and *p*-values. *F*- and *p*-values of excluded factors were taken from full models. Effects sizes are given for terms that remained in the final model. The results shown originate from S models; results written in italics originate from models containing the fixed factor “person”.

Factor	df	*F*	*p*	Factor level	Effect size
Final model	7	42.482	<0.001		

Species	1	2.043	0.153	Crow	1.421
				Raven	−0.348

Familiarity	1	80.397	<0.001	Long-term	−0.954
				Short-term	0

Season	2	72.846	<0.001	Pre-breeding	−1.765
				Breeding	0.976
				Post-breeding	0

Species × familiarity	1	3.961	0.047	Crow × long-term	0.745
				Crow × short-term	0
				Raven × long-term	0
				Raven × short-term	0

Season × familiarity	2	49.158	<0.001	Pre-breeding × long-term	2.958
				Breeding × long-term	2.568
				Post-breeding × long-term	0
				Pre-breeding × short-term	0
				Breeding × short-term	0
				Post-breeding × short-term	0

*Person (familiarity)*	*11*	*17.241*	*<0.001*	*A*	*2.384*
				*B*	*−0.421*
				*C*	*−0.812*
				*D*	*1.972*
				*E*	*0*
				*D*	*−2.966*
				*F*	*−1.955*
				*G*	*0.148*
				*H*	*0.349*
				*I*	*−0.749*
				*J*	*−0.824*
				*K*	*−0.972*
				*L*	*0*
