# Supplementary material for: Familiarity with the experimenter influences the performance of Common ravens (Corvus corax) and Carrion crows (Corvus corone corone) in cognitive tasks
Source: Behav Processes. 2014 Mar;103(100):129–37. doi: 10.1016/j.beproc.2013.11.013 (PMC4003535; doi:10.1016/j.beproc.2013.11.013)
Supplement: Supplementary file 1 [file mmc1.docx]

Table S1: Functional context and descriptions of affiliative, stress- related and comfort behaviours observed in the birds with different experimenters. The behaviours preen, pluster, stretch and scratch were summarised as “comfort behaviour” for the analysis. Behaviours marked with (f) were measured as frequencies, behaviours marked with (d) as durations.

| **affiliative behaviours** | approach (f) | The bird moves towards the experimenter so that the distance between the bird and the wire mesh in front of the experimenter is reduced to less than 50cm. |
| --- | --- | --- |
| **stress-related behaviours** | wing quivering (f) | The bird repeatedly moves its shoulders forwards and back again in a quick twitching movement. The wings are kept close to the body. |
|  | fluttering (d) | The bird repeatedly flies short distances, stopping in between for up to 30 seconds. No goal-oriented behaviours are seen during the short stop intervals. |
| **comfort behaviours** | preen (d) | The bird moves its beak into its plumage and then moves the feathers trough its beak. |
|  | pluster (d) | The bird erects the feathers of its body. |
|  | stretch (d) | The bird moves one leg away from its body and straightens it. The wing on the same side of the body may also be straightened. |
|  | scratch (d) | The bird moves the claws of one foot through its plumage quickly. |

**Table S2:** Results of GLMMs with performance in the object choice task as the dependent variable. Factors entered into the model, degrees of freedom (df), F- and p-values were taken from full models. The results shown originate from S models; results marked grey originate from models containing the fixed factor “person”.

| **Factor** | **df** | **F** | **P** |
| --- | --- | --- | --- |
| Final Model | 7 | 0.950 | 0.466 |
| Species | 1 | 0.056 | 0.813 |
| Familiarity | 1 | 0.031 | 0.861 |
| Season | 2 | 1.059 | 0.347 |
| Species*Familiarity | 1 | 0.093 | 0.761 |
| Season*Familiarity | 2 | 1.156 | 0.315 |
| Person (Familiarity) | 11 | 1.242 | 0.253 |

**Table S3:** Results of GLMMs with approach behaviour as the dependent variable. Statistical parameters for the final model are given as well as factors entered into the model, degrees of freedom (df), F- and p-values. F- and p-values of excluded factors were taken from full models. Effects sizes are given for terms that remained in the final model. The results shown originate from S models; results marked grey originate from models containing the fixed factor “person”.

| **Factor** | **df** | **F** | **P** | **Factor Level** | **Effect size** |
| --- | --- | --- | --- | --- | --- |
| Final Model | 4 | 7.458 | < 0.001 |  |  |
| Species | 1 | 6.706 | 0.010 | crow | -1.337 |
|  |  |  |  | raven | 0 |
| Familiarity | 1 | 10.107 | 0.002 | long-term | 3.172 |
|  |  |  |  | short-term | 0 |
| Species*  Familiarity | 1 | 3.643 | 0.058 | crow*long-term | -1.924 |
|  |  |  |  | crow*short-term | 0 |
|  |  |  |  | raven*long-term | 0 |
|  |  |  |  | raven*short-term | 0 |
| Season | 2 | 1.378 | 0.254 |  |  |
| Season*  Familiarity | 3 | 4.855 | 0.003 | pre-breeding*long-term | -3.474 |
|  |  |  |  | breeding*long-term | 0 |
|  |  |  |  | pre-breeding*short-term | -0.302 |
|  |  |  |  | breeding*short-term | -1.069 |
|  |  |  |  | post-breeding*short-term | 0 |
| Person  (Familiarity) | 10 | 0.942 | 0.495 |  |  |

Table S4: Results of GLMMs with comfort behaviour as the dependent variable. Factors entered into the model, degrees of freedom (df), F- and p-values are given. F- and p-values of excluded factors were taken from full models. Effects sizes are given for terms that remained in the final model. The results shown originate from S models; results marked grey originate from models containing the fixed factor “person”.

| **Factor** | **df** | **F** | **P** | **Factor Level** | **Effect size** |
| --- | --- | --- | --- | --- | --- |
| Final Model | 3 | 7.006 | < 0.001 |  |  |
| Species | 1 | 20.525 | < 0.001 | crow | -1.907 |
|  |  |  |  | raven | 0 |
| Familiarity | 1 | 1.222 | 0.270 |  |  |
| Season | 2 | 3.271 | 0.040 | pre-breeding | 0.969 |
|  |  |  |  | breeding | -0.022 |
|  |  |  |  | post-breeding | 0 |
| Species*  Familiarity | 1 | 0.003 | 0.959 |  |  |
| Season*  Familiarity | 1 | 0.003 | 0.953 |  |  |
| Person  (Familiarity) | 10 | 1.604 | 0.107 |  |  |

**Table S5:** Results of GLMMs with stress-related behaviour as the dependent variable. Factors entered into the model, degrees of freedom (df), F- and p-values were taken from full models. The results shown originate from S models; results marked grey originate from models containing the fixed factor “person”.

| **Factor** | **df** | **F** | **P** | **Factor Level** | **Effect size** |
| --- | --- | --- | --- | --- | --- |
| Final Model | 1 | 0.221 | 0.639 |  |  |
| Species | 1 | 0.000 | 0.990 |  |  |
| Familiarity | 1 | 0.221 | 0.639 | long-term | -0.255 |
|  |  |  |  | short-term | 0 |
| Season | 2 | 0.365 | 0.649 |  |  |
| Species*Familiarity | 1 | 0.000 | 1.000 |  |  |
| Season*Familiarity | 1 | 0.658 | 0.418 |  |  |
| Person  (Familiarity) | 10 | 0.995 | 0.449 |  |  |
